# Exploring water-absorbing capacity: a digital image analysis of seeds from 120 wheat varieties

**DOI:** 10.1038/s41598-024-57193-w

**Published:** 2024-03-21

**Authors:** Tooba Khan, Muhammad Jamil, Aamir Ali, Sana Rasheed, Asma Irshad, Muhammad Faisal Maqsood, Usman Zulfiqar, Talha Chaudhary, M. Ajmal Ali, Mohamed S. Elshikh

**Affiliations:** 1 Department of Botany, The Islamia University of Bahawalpur, Bahawalpur, Pakistan; 2 Department of Botany, University of Sargodha, Sargodha, Pakistan; 3 Department of Agronomy, Faculty of Agriculture and Environment, The Islamia University of Bahawalpur, Bahawalpur, 63100 Pakistan; 4 Faculty of Agricultural and Environmental Sciences, Hungarian University of Agriculture and Life Sciences, 2100 Godollo, Hungary; 5 Department of Botany and Microbiology, College of Science, King Saud University, 11451 Riyadh, Saudi Arabia

**Keywords:** Digital image analysis, Wheat seeds, Wheat genotypes, Imbibition, Shape, Size, SmartGrain, Plant sciences, Plant physiology

## Abstract

Wheat is a staple food crop that provides a significant portion of the world's daily caloric intake, serving as a vital source of carbohydrates and dietary fiber for billions of people. Seed shape studies of wheat typically involve the use of digital image analysis software to quantify various seed shape parameters such as length, width, area, aspect ratio, roundness, and symmetry. This study presents a comprehensive investigation into the water-absorbing capacity of seeds from 120 distinct wheat lines, leveraging digital image analysis techniques facilitated by SmartGrain software. Water absorption is a pivotal process in the early stages of seed germination, directly influencing plant growth and crop yield. SmartGrain, a powerful image analysis tool, was employed to extract precise quantitative data from digital images of wheat seeds, enabling the assessment of various seed traits in relation to their water-absorbing capacity. The analysis revealed significant transformations in seed characteristics as they absorbed water, including changes in size, weight, shape, and more. Through statistical analysis and correlation assessments, we identified robust relationships between these seed traits, both before and after water treatment. Principal Component Analysis (PCA) and Agglomerative Hierarchical Clustering (AHC) were employed to categorize genotypes with similar trait patterns, providing insights valuable for crop breeding and genetic research. Multiple linear regression analysis further elucidated the influence of specific seed traits, such as weight, width, and distance, on water-absorbing capacity. Our study contributes to a deeper understanding of seed development, imbibition, and the crucial role of water absorption in wheat. These insights have practical implications in agriculture, offering opportunities to optimize breeding programs for improved water absorption in wheat genotypes. The integration of SmartGrain software with advanced statistical methods enhances the reliability and significance of our findings, paving the way for more efficient and resilient wheat crop production. Significant changes in wheat seed shape parameters were observed after imbibition, with notable increases in area, perimeter, length, width, and weight. The length-to-width ratio (LWR) and circularity displayed opposite trends, with higher values before imbibition and lower values after imbibition.

## Introduction

Wheat is a vital cereal grain widely cultivated for its edible kernels, playing a crucial role in global food stability. It serves as a primary staple for billions in North America, Europe, and Asia, offering essential nutrients like carbohydrates, fiber, protein, vitamins, and minerals. In this study, a high-capacity phenotyping system called SmartGrain Version 1.1(2012/8/1) was developed to assess seed morphology using image analysis. SmartGrain autonomously identifies and outlines all seeds in a digital image and calculates various metrics, including length, width, seed area, perimeter length, and additional parameters^1^. Digital image analysis (DIA) converts digital images of plant organs into numerical measurements, facilitating rapid and large-scale data generation. DIA methods measure object dimensions and shape using software like ImageJ, especially valuable in assessing traits like milling quality and yield in crops like wheat. Geometric models suggest that wheat seed shape can impact flour yield by maximizing endosperm-to-bran ratio, offering economic insights^2^. Braadbaart & Van Bergen,^3^ also examines the size and shape and shape of fruits and seeds by using digital imaging for precise measurements, and the goal is to analyze size and shape changes in heated modern fruits and seeds and identify the underlying factors.

SHAPE software is used to analyze seed shape by utilizing photographs of seeds in both vertical and horizontal orientations. It employs elliptic Fourier descriptors to capture both 2D and 3D characteristics, offering a comprehensive approach to seed shape analysis. GrainScan offers a high-throughput approach for efficient and precise measurements of cereal grain size and color^4^. Wheat grain's physical appearance and morphology can be influenced by its moisture content. While moisture content doesn't directly affect grain quality, it can indirectly reduce storage duration for grains exceeding recommended moisture levels^5^. Image processing techniques were used to determine the lengths, widths, thickness, environment, and shape coefficients of the wheat grains was performed by Sabanci et al.^6^. Seeds experience volume and shape changes during germination, especially when they start with a near-spherical, symmetric shape. These changes involve irregular alterations, with length increasing more than width and thickness. Studies on seed mass are common, but research on seed volume, density, and porosity during wetting is relatively limited, even for dry state seed characteristics^7^. Recently, a high-throughput approach has been employed to capture the variations in grain size and shape across numerous mapping populations, elite varieties, and a diverse collection of ancestral wheat species^8^. Digital image (DI) technique allows for the digital measurement of changes in seed shape parameters caused by heat stress and DI encompasses the conversion of digitally captured images into numerical values^9^. Image analysis enhances the understanding of germination by enabling non-invasive monitoring of dimensional changes over time, eliminating the need to handle germinated seeds. This technology offers valuable insights into the germination process^10^. Various parameters related to the shape, size, color, and texture of bean seeds was performed by Kapadia,^11^. Image analysis has proven to be a valuable tool for monitoring seed germination. Another widely used method for shape analysis of plant organs, including seeds, is the elliptic Fourier descriptors introduced by Kuhl and Giardina^12^. Geometrical models have demonstrated that alterations in grain size and shape can potentially lead to flour yield improvements of up to 5%^13^. Grain weight is determined by several factors, including grain length, width, and area. These traits are stably inherited and exhibit higher heritability compared to overall yield^14^.

The germination process is characterized by seed water absorption and is commonly described as consisting of three distinct phases early imbibition, volume and mass changes in the seeds and proper germination^15^. Cabral et al.^16^ said that the size and shape of wheat kernels have a direct impact on kernel weight and test weight, as well as influencing grain protein content and milling yield. Goriewa-Duba et al.^17^ performed an experiment on the utilization of digital image analysis has greatly facilitated various processes in the field of plant phenotyping, initially, this analytical technique was employed to verify the identity of wheat kernels by examining their shape and color characteristics. Digital image processing techniques, often coupled with multivariate statistical analysis, yields authentic outcomes in wheat variety recognition by Alemu,^18^. Seed size plays a significant role in determining seed quality and has a direct impact on the growth and establishment of seedlings^19^. Kumar et al.^20^ work on the Grain shape and size that have a direct impact on both wheat quality and grain yield. Digital imaging analysis (DIA) is a method used to convert digital images into quantitative measurements, allowing for the generation of large sets of quantitative data^21^. GrainScan, a software package designed for high-throughput phenotyping of cereal grains, was used to collect digital image measurements. Grain length (GL) and grain width (GW) were measured using the default-automated threshold, and the mean value of 20 seeds per replicate was recorded^22^. A classification criteria based on grain shape variations has been developed for barley, wheat, and rice, aiming to improve pattern recognition through image processing techniques^23^. Digital imaging provides measurements such as area, perimeter, length and width, which are used to define seed dimensions. It can also capture additional characteristics like seed thickness, asymmetric skewing, and roughness, contributing to change in seed shape^24^. The novelty of the research in wheat seed shape analysis is found in its specific focus on wheat genotypes, the consideration of imbibition-induced changes, the utilization of advanced automated analysis tools like SmartGrain, and the incorporation of comprehensive statistical techniques to unravel the complex relationships between seed traits and their impact on overall variability and physiological processes. So, this study was planned with the objectives to evaluate the effect of water absorption on wheat and to determine the effect of seed imaging in different wheat varieties.

## Materials and methods

To assess the impact of imbibition on the morphology and dimensions of wheat seeds, specimens from 120 bread wheat genotypes were collected from the department of plant sciences, Quaid-i-Azam University Islamabad, Pakistan with the due permission. The objective is to determine whether there is a correlation between the seed size and shape and their water absorbing ability. The experiment will involve measuring the length, width, and thickness of the wheat seeds, and then analyzing the data to determine whether there is a relationship between the morphometric properties and the water absorbing ability of the seeds.

### Experimental design

In this experiment 120 genotypes of wheat elite lines from diverse parentage back ground was taken. 16 seeds of each genotype with three replicates were weighted and placed on the black board with specific arrangement. Crease side of seed must be down on board. By using Sony a99 CAMERA we capture the image from 17 cm distance in preserved manner that show the dark back ground. And now place these seeds in petri dishes containing filter paper in previous arrangement. Give 10 ml water to each petri dish and leave the seeds for 14–16 h. Now placed the seeds after imbibition on black board and again capture the image. All the seeds in each petri dish were weighted before imbibition and after imbibition. The final weight was the 16-seed weight after water uptake and initial weight was the original 16-seed weight before water uptake. This experiment repeated for each genotype.

### Image acquisition

**Figure 1:**
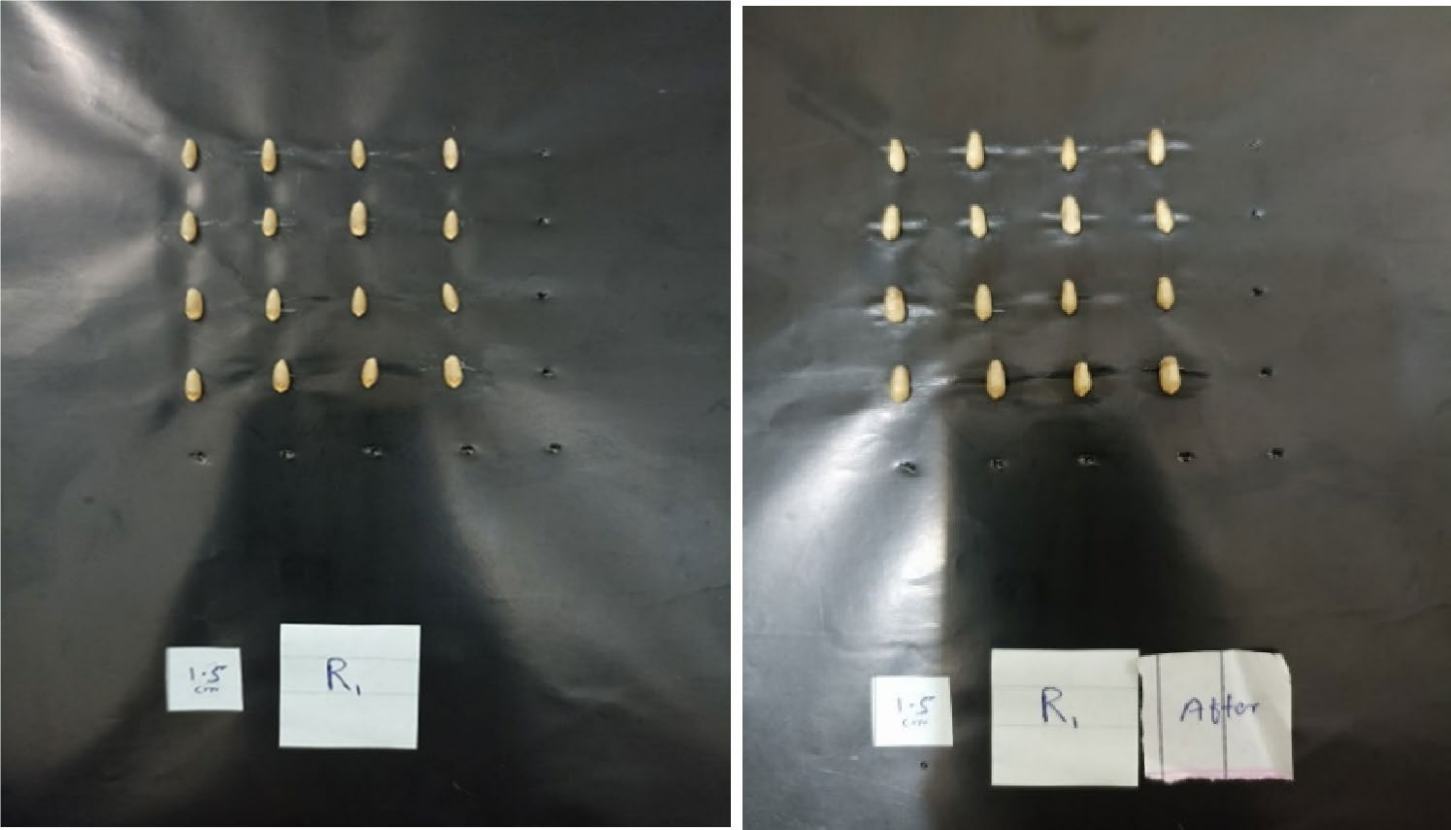
Wheat seed images of one genotype before and after imbibition.

Preprocess the acquired images to enhance the quality and remove any unwanted artifacts. This may include techniques such as image sharpening, or contrast adjustment. Segment the wheat grains from the background using image segmentation algorithms. This step aims to separate individual grains for further analysis. Establish an imaging system capable of capturing high-resolution images prior to seed soaking. Arrange the seeds beneath the imaging system, ensuring consistent lighting conditions and a stable setup. Use a high-resolution digital camera or scanner to capture images of individual seeds. Ensure consistent lighting and positioning during image capture. The images will be analyzed by using the software *SmartGrain*^1^. This software automatically recognizes all seeds within a digital image (Fig. 1), detect outline and then calculates these parameters; Length (L),Width (W), Seed Area (AS), Perimeter length (PL), Length to width ratio (LWR), Circularity (CS), and Distance (DS).

### Statistical analysis

The investigation involved utilizing SmartGrain software to extract shape analysis results, encompassing parameters like seed length, width, area, perimeter, and others, from each wheat seed genotype. The collected data underwent organization for subsequent statistical analysis. Descriptive statistics, including mean, median, standard deviation, minimum, maximum, and quartiles, were computed to provide a comprehensive overview of seed shape characteristics. Furthermore, statistical hypothesis tests were conducted to identify significant differences in seed shape parameters across various genotypes or experimental conditions. Depending on data distribution and research objectives, tests such as t-tests and ANOVA were applied. For statistical analyses, software packages such as Minitab-19 was used for descriptive statistics, ANOVA, PCA and regression analysis. User-friendly interface of Minitab-19 software makes it an accurate choice for efficient descriptive statistical analysis. Origin-22 software was used to create boxplot and cluster analysis. Due to its customizability, the Origin software was used to generate box plots to present data distribution; additionally, due to its robustness, in order to easily and clearly understand the grouping pattern, cluster analysis was also performed using this software. IPASTIC^25^ for various indices, RStudio for correlation and heatmaps. Due to its unique feature of simultaneously offering a combination of indices, iPASTIC, an online toolkit, was used to compute indices for identifying and ranking top-performing genotypes. Due to the ability to produce wide range of publication-quality multi-colored visualizations facilitating the exploration of complex data patterns, we used statistical packages in R-software because it becomes a preferable choice when generating heatmaps of correlation and classification of rows (genotypes) and columns (traits).

### Plant guidelines

All the plant experiments were performed by following relevant institutional, national, and international guidelines and legislations.

### Permissions

Permissions were obtained to use the seed for research purposes.

## Results

In this study, we employed the SmartGrain software to investigate the imbibition-induced changes in wheat grain seed shape. By leveraging the capabilities of SmartGrain, we aimed to quantitatively assess alterations in key seed shape parameters, including area, perimeter, length, width, circularity, and diameter, as seeds imbibed water.

**Figure 2:**
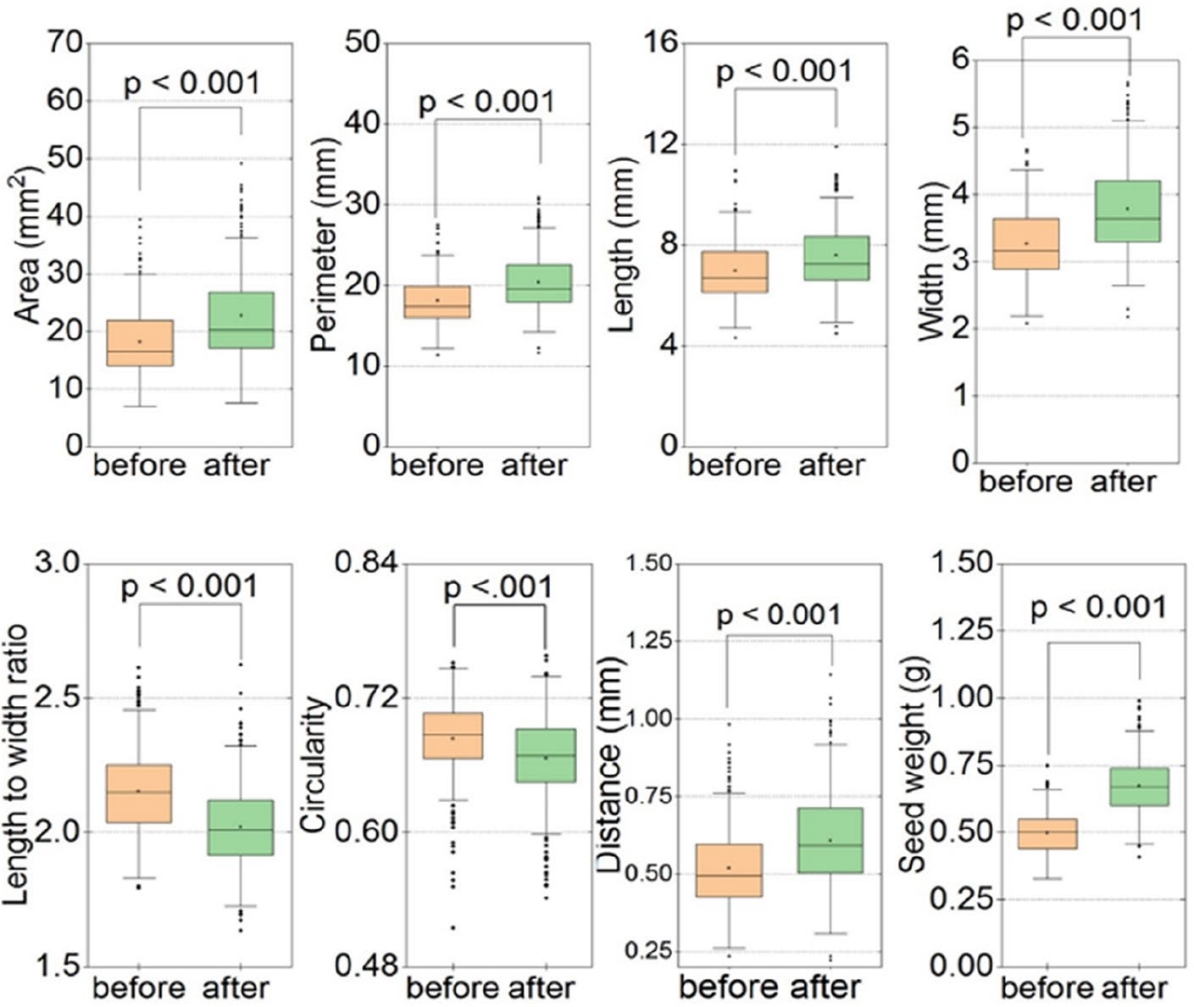
Boxplots of eight studied traits comparing before and after seed treatment.

Before imbibition, the box plot (Fig. 2) indicates that the seed area, perimeter, length, width, distance, and weight exhibit lower values, whereas higher values were shown after imbibition. Conversely, the LWR (length-to-width ratio) and circularity display higher values before imbibition and lower values after imbibition.

The Table 1 presents descriptive statistics for various seed parameters in a wheat analysis, including means, standard errors (SE), minimums, maximums, and quartile values (Q1 and Q3), along with paired t-test results for the differences between "after" and "before" treatments. The average weight of seeds after treatment is 0.68 g, with a standard error of 0.01.Seeds' weight ranged from 0.41 g (minimum) to 0.99 g (maximum).The first quartile (Q1) is 0.60 g, the median is 0.67 g, and the third quartile (Q3) is 0.74 g. The weight increased by 35.47% after treatment.

**Table 1:** Descriptive statistics of seed parameters along with paired t-test.

Variable	Treatment	Mean	SE	Min	Max	Q1	Median	Q3	Diff (%)	t-value	d
Weight (g)	After	0.68	0.01	0.41	0.99	0.60	0.67	0.74	35.47	65.61	3.46
	Before	0.50	0.00	0.33	0.75	0.44	0.50	0.55			
A (mm^2^)	After	22.68	0.40	7.68	49.15	17.23	20.20	26.86	24.49	19.90	1.05
	Before	18.22	0.31	7.06	39.47	14.02	16.53	22.02			
P (mm)	After	20.41	0.18	11.64	30.90	17.90	19.53	22.56	13.04	23.30	1.22
	Before	18.06	0.15	11.33	27.52	15.97	17.41	19.92			
L (mm)	After	7.60	0.07	4.49	11.91	6.64	7.24	8.35	8.81	16.75	0.88
	Before	6.98	0.06	4.33	10.97	6.14	6.72	7.75			
W (mm)	After	3.79	0.03	2.18	5.66	3.30	3.64	4.21	15.78	26.32	1.39
	Before	3.27	0.03	2.09	4.67	2.90	3.16	3.64			
LWR	After	2.02	0.01	1.64	2.63	1.92	2.01	2.12	− 6.00	18.72	0.99
	Before	2.15	0.01	1.80	2.61	2.04	2.15	2.25			
CS	After	0.67	0.00	0.54	0.76	0.65	0.67	0.69	− 2.56	9.53	0.50
	Before	0.68	0.00	0.52	0.75	0.67	0.69	0.71			
DS (mm)	after	0.61	0.01	0.22	1.14	0.50	0.59	0.71	17.07	10.18	0.54
	Before	0.52	0.01	0.24	0.98	0.43	0.50	0.60			

*SE* Standard error, *Min* Minimum, *Max* Maximum, *Q* 25th percentile, *Q3* 75th percentile, *Diff (%)* Percentage of difference, *d* Cohen’s d (Effect size).

The t-value for the paired t-test is 65.61, indicating a significant difference (p < 0.05). Cohen's d (effect size) is 3.46, indicating a large effect. The descriptive statistics provide insights into how various seed parameters of wheat changed after treatment. The paired t-test results suggest significant differences between the "after" and "before" treatments for most parameters, with effect sizes ranging from small to large.

The Table 2 presents the breakdown of percentage variation in seed traits explained by different factors and sources of variation. Variety (V) factor represents 120 wheat varieties or types of seeds used in the study. For example, the variation in weight was explains 41.80% by the varieties and 48.76% by the treatments; their interactive (V × T) effect explained very little (0.38%) proportion of overall variation in seed weight. Coefficient of variation (CV%) for the entire ANOVA model, indicating the overall variability in comparison to grand mean for all studied traits.

**Table 2:** Two factor full factorial analysis of variance (ANOVA) of seed shape traits.

Trait (unit)	Code	Variety (V)	Treatment (T)	V × T	Error	GM	CV%
Weight (g)	WT	41.80	48.76	4.38	5.06	0.59	5.94
Area (mm^2^)	A	75.05	9.76	6.46	8.73	20.45	12.64
Perimeter (mm)	P	73.09	12.04	5.16	9.71	19.24	6.73
Length (mm)	L	79.77	5.99	4.87	9.36	7.29	6.46
Width (mm)	W	67.60	16.74	5.91	9.74	3.53	6.83
Length-to-width-ratio	LWR	60.81	14.56	8.89	15.74	2.09	3.94
Circularity	CS	59.62	5.59	11.63	23.16	0.68	3.23
Distance (mm)	DS	47.00	8.46	17.48	27.05	0.56	17.22

Variation explained by three known sources of variation (V, T, VxT) was significant (p-value < 0.001) in all studied traits. GM is grand mean and CV% is percentage of coefficient of variation of the overall analysis of variance (ANOVA) model.

Top ten genotypes ranked on the basis of 11 iPASTIC indices are given in Table 3 before and after imbibition. These indices were calculated through an online software tool^25^ using seed weight of before and after water application. Rank column indicates ranking of the wheat genotypes based on their overall performance according to the 11 iPASTIC indices^25^. Lower ranks generally indicate better performance in relation to the difference of seed weight before and after water absorption.

**Table 3:** Ten-promising genotypes (with studied traits) ranked on the basis of 11 iPASTIC indices.

Rank	Variety	Before	After	WA	wt	A	P	L	W	LWR	CS	DS
1	v112	0.62	0.72	0.10	0.67	16.67	17.17	6.56	3.25	2.03	0.71	0.58
2	v120	0.63	0.78	0.16	0.71	18.23	17.92	6.72	3.56	1.90	0.71	0.49
3	v102	0.66	0.83	0.18	0.75	17.01	17.19	6.58	3.33	1.99	0.71	0.54
4	v9	0.57	0.71	0.14	0.64	23.52	20.27	7.62	3.96	1.94	0.71	0.58
5	v113	0.61	0.78	0.17	0.69	17.56	17.40	6.64	3.39	1.96	0.72	0.50
6	v8	0.59	0.75	0.16	0.67	25.46	21.31	7.86	4.13	1.92	0.70	0.48
7	v118	0.58	0.73	0.15	0.65	20.53	18.92	6.99	3.76	1.86	0.72	0.49
8	v98	0.62	0.82	0.20	0.72	35.89	25.36	9.75	4.68	2.10	0.69	0.78
9	v18	0.67	0.91	0.23	0.79	24.94	21.43	8.25	3.99	2.07	0.70	0.61
10	v107	0.56	0.65	0.09	0.61	16.66	17.31	6.56	3.24	2.04	0.70	0.58

*Before and after* 16-seed weight (g) before and after water absorbed, *WA* water absorbed (ml), *wt* average seed weight (g) of before and after; other trait legends are same as in Table 1.

In the Table 3, wt is the mean value before and after weight. WA is the water absorbed before and after imbibition. ‘P’ Perimeter is the length of the outer boundary of the seed, and it can indicate the complexity of the seed shape. L and W is the length and width of the seed in millimeters. LWR is a ratio that indicates whether the seed is more elongated or compact, with higher values suggesting greater elongation which reduced after the water absorbed.

Circularity measures how closely the seed shape resembles a perfect circle, with higher values indicating a more circular shape. DS may represent a measure of the distance between specific points on the seed.

**Figure 3:**
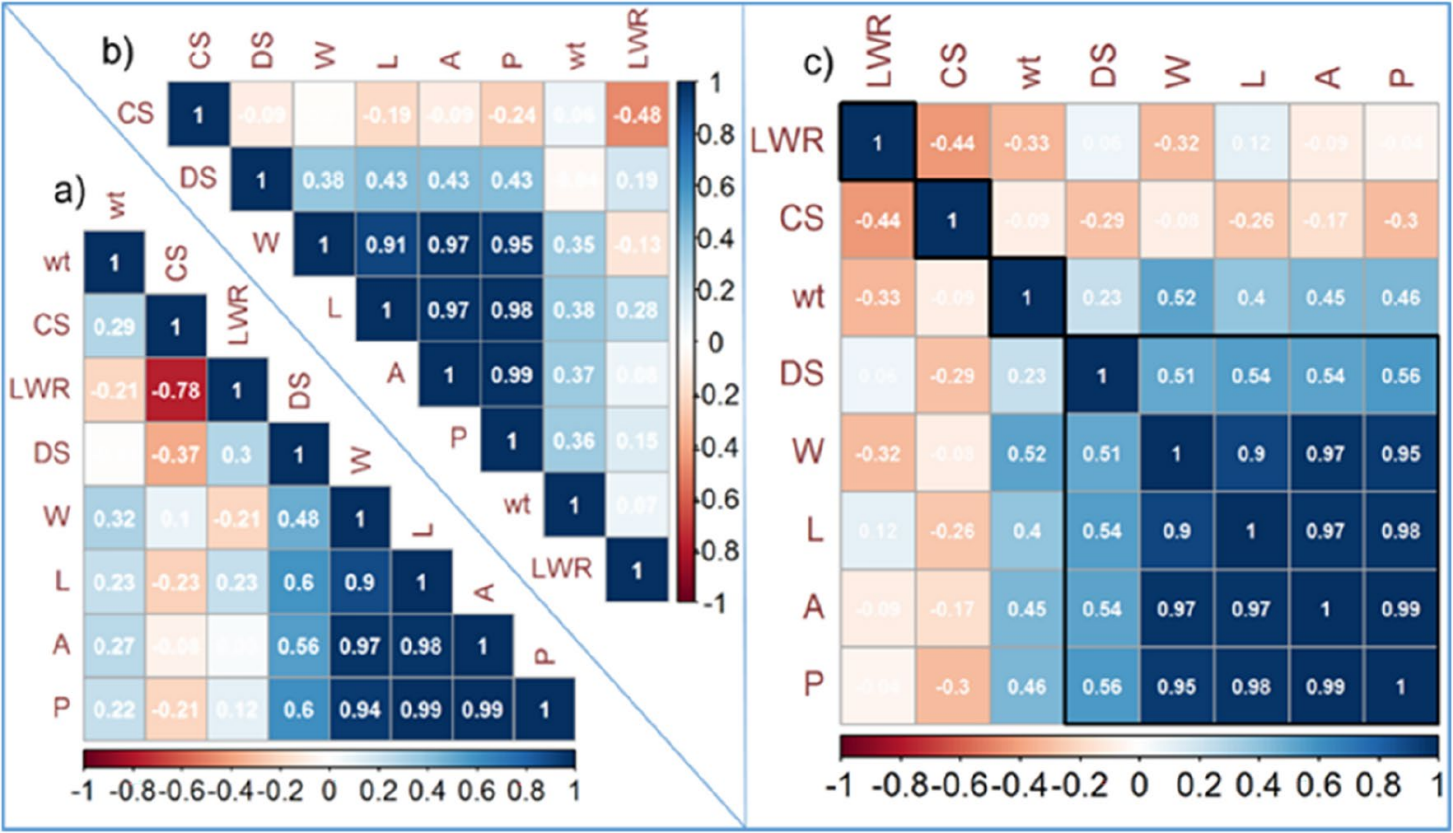
Pearson’s correlation coefficient (r) among studied traits before (n = 120) the water treatment (**a**), after (n = 120) the water treatment (**b**) and combined (n = 240) observations (**c**). Traits have been ordered by agglomerative hierarchical clustering (AHC) method and the absolute critical r-value was calculated as ± 0.212 beyond which the correlation was considered significant (p < 0.01) at 118 degree of freedom for (**a**) and (**b**), while in case of (**c**) r-value ± 0.212 was significant (p < 0.001) at 238 degree of freedom.

The pattern of correlation among the studied seed shape parameters before (Fig. 3a) and after the water treatment (Fig. 3b) elucidates the importance of seed circularity that is positively correlated with seed weight before the water treatment and after that becomes negatively linked with DS.

Before Water Treatment (n = 120) Pearson's correlation coefficients are calculated among the studied traits for a dataset of 120 observations taken before the water treatment. To determine if the correlations are statistically significant, a critical r-value of ± 0.212 is set, and correlations beyond this threshold are considered significant at a 118 degrees of freedom with a significance level of p < 0.01.After Water Treatment (n = 120) Pearson's correlation coefficients are calculated for the same set of studied traits, but this time based on 120 observations taken after the water treatment. Again, a critical r-value of ± 0.212 is used, and correlations exceeding this threshold are considered significant at 118 degrees of freedom with a significance level of p < 0.01.

Combined Observations (n = 240) In this case, Pearson's correlation coefficients are calculated for the combined dataset, which includes both the before and after water treatment observations, totaling 240 data points. The critical r-value of ± 0.212 remains the same, but this time the correlations are considered significant at a higher level of significance, p < 0.001, and are tested with 238 degrees of freedom.

**Figure 4:**
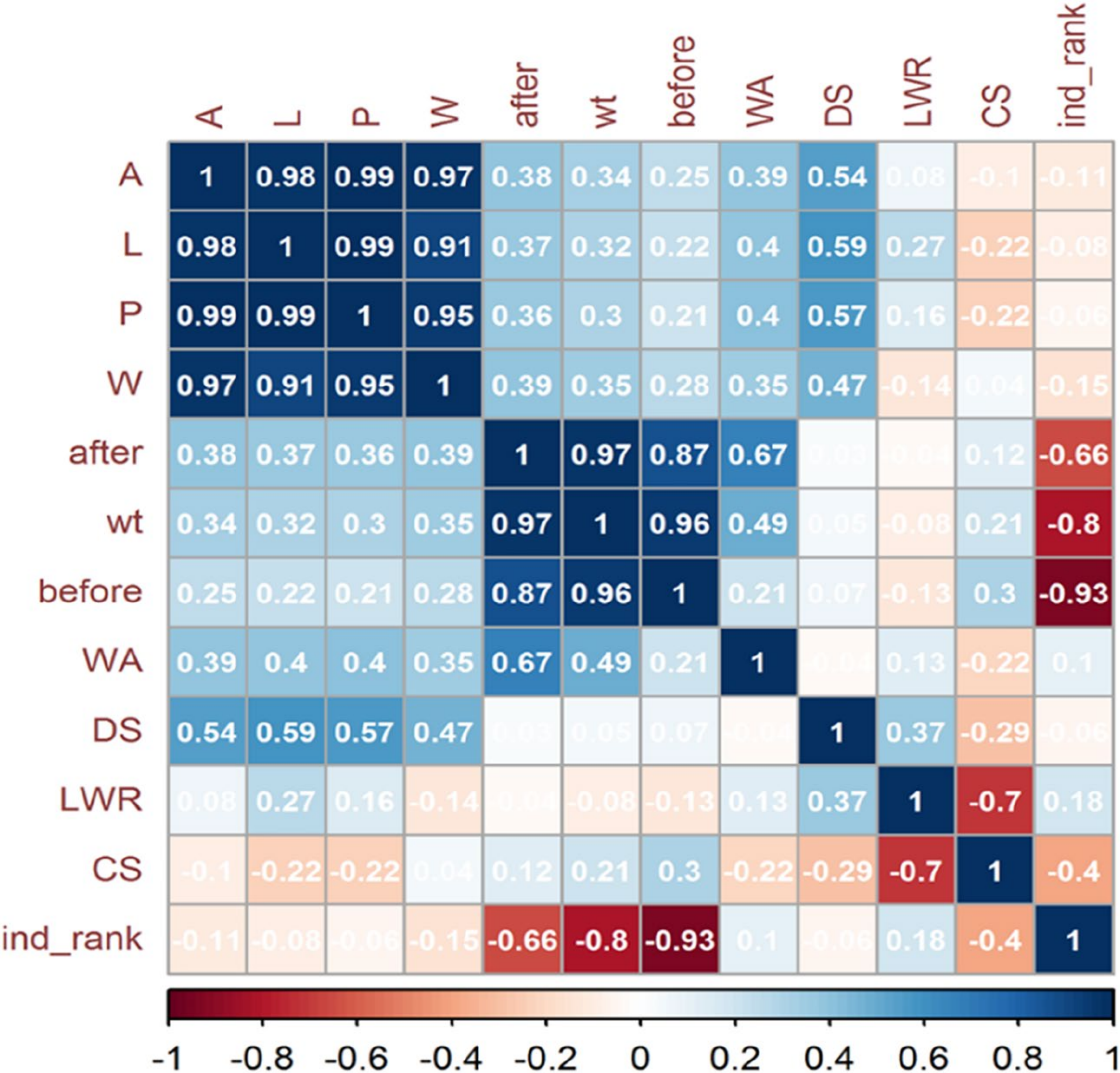
Pearson’s correlation coefficient (r) among twelve traits, each with 120 observations. Traits have been ordered according to first principle component (FPC) method and the absolute critical r-value was calculated as ± 0.297 beyond which the correlation was considered significant (p < 0.001) at 118 degree of freedom. Trait legends are same as in Table 1.

A hierarchical clustering analysis (AHC) has categorized 12 variables. There are 66 pair wise correlations among 12 traits (Fig. 4). The area exhibits a highly significant positive correlation (p < 0.001) of 0.98 with length, while its correlation with perimeter and width is also highly significant at the 0.001 level, measuring 0.99 and 0.97, respectively. The area along the after, wt, before, WA and DS are also significant and the strength of this correlation is 0.38, 0.34, 0.25, 0.39 and 0.54 respectively. LWR, CS and ind_rank show no correlation with area. The length are in significantly (p < 0.001) positive correlation with P, W, after, wt, before, WA and DS. The strength of correlation in these variables is 0.99, 0.91, 0.37, 0.32, 0.22, 0.4 and 0.59 respectively. The variable before is significantly (p < 0.001) positive correlated with CS (0.3) and negatively correlated with ind_rank (-0.93).WA shows no correlation. When we discuss the trait DS, it also shows the significance positive correlation with LWR (0.37) and negative correlation with CS (-0.29). LWR and CS shows no correlation. The p is for probability value and the very low p-value of less than 0.001 (p < 0.001) indicates strong evidence against the null hypothesis. It suggests that the observed correlation is highly unlikely to have occurred by chance alone. Therefore, you would typically reject the null hypothesis and conclude that there is a statistically significant correlation between the variables being analyzed.

**Figure 5:**
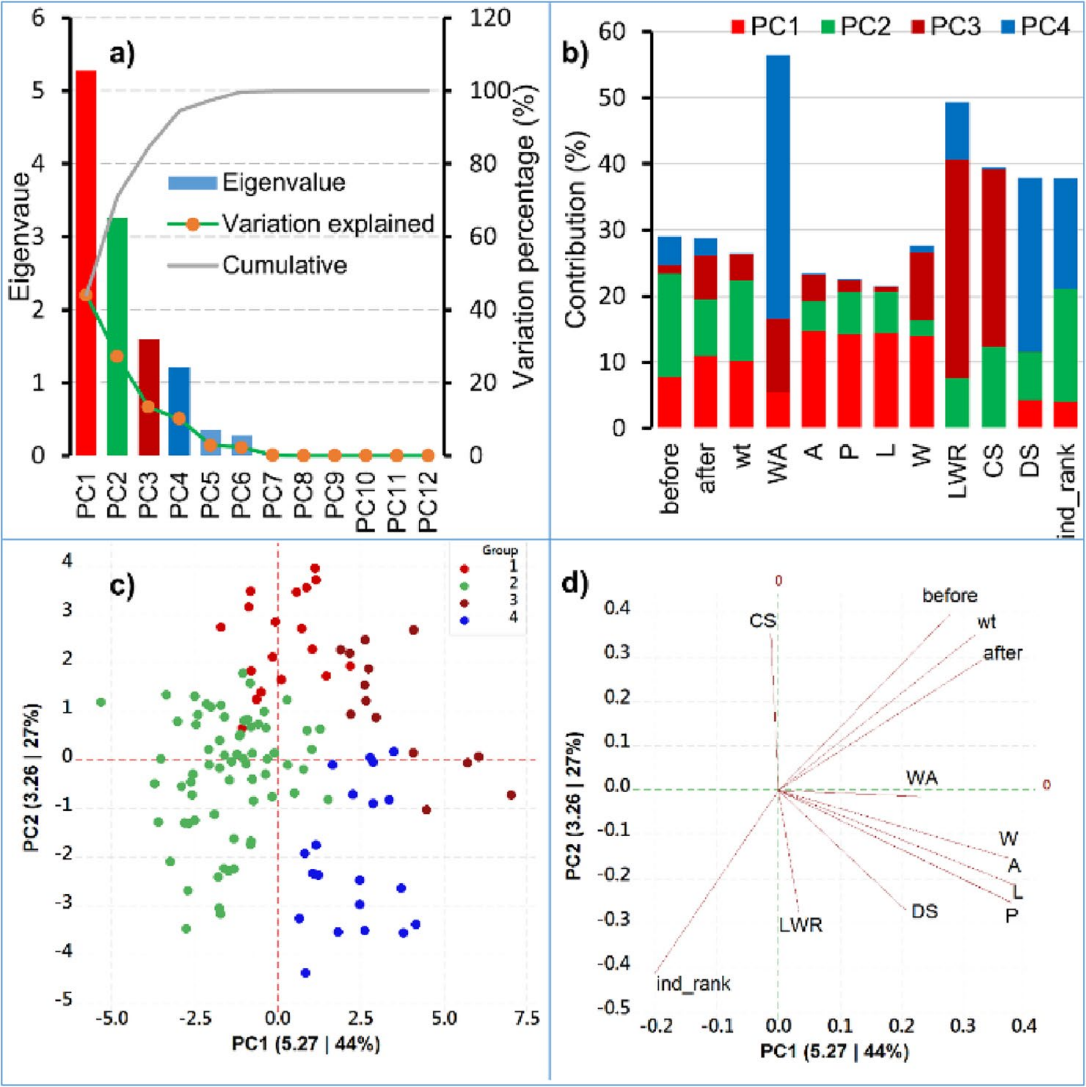
Principal component analysis (PCA) of twelve traits with 120 genotypes showing principal components (PC) with eigenvalues and variation percentage explained by each PC (**a**), contribution of each trait to potential PCs (**b**), score plot of 120 observations as revealed by first principal component (PC1) along x-axis with eigenvalue (5.27) and variation explained (44%) by PC1. Perpendicular to PC1, along y-axis is the second principal component (PC2) having eigenvalue (3.26) and variation explained (27%) by PC2 (**c**). Loading plot of first two components (**d**) is showing two dimensional spread of variables as vectors; trait legends are same as in Table 1.

As a result of eigenvalue analysis, Fig. 5a illustrates the relationship between PCA components, their corresponding eigenvalues, and the percentage of variation they explain. PC1 has an eigenvalue of 5.27, explaining 44% of the total variation, followed by PC2 with an eigenvalue of 3.26 (27% variation). PC3 and PC4 have eigenvalues of 1.595 (13.3%) and 1.2111 (10.1%), respectively. The cumulative variance of the first four PCs with the eigenvalues above one is 94.6%. Principal components with eigenvalues up to 1 are considered meaningful, providing insights into the amount of information captured from the original data.

In the Fig. 5b, individual traits contributing to each of the first four principal components have been highlighted. Each trait can be thought of as having a weight or influence on the formation of the PCs. The contribution of each trait to potential PCs helps us understand which traits are most relevant in explaining the underlying patterns of variation in the data. The preceding data presents four principal components: in variable ‘before’ PC1 with a value of 7.78, PC2 with 15.60, PC3 with 1.28, and PC4 with 4.37. In Area, perimeter and length PC1 is influenced by area with a value of 14.6, perimeter with 14.29, and length with 14.36. Meanwhile, PC2, within this trio of variables (A, P, L), is associated with values of 4.49, 6.35, and 6.35, respectively. PC3 is characterized by values of 4.12 for 'A' (area), 1.82 for 'P' (perimeter), and 0.67 for 'L' (length). PC4 exhibits values of 0.18 for 'A,' 0.09 for 'P,' and 0.05 for 'L'. The score plot (Fig. 5c) displays the distribution of 120 genotypes across the first and second principal components (PC1 and PC2) obtained from PCA and all 120 genotypes are classified into four clusters. PC1 (eigenvalue 5.27) explains 44% of the variation, and PC2 (eigenvalue 3.26) explains 27%. The plot, utilizing color coding, helps visualize genotype distinctions and connections in the reduced-dimensional space, facilitating analysis and interpretation. The loading plot illustrates how the twelve original traits contribute to the first two principal components (PC1 and PC2) through vectors in a two-dimensional space. The length and direction of these vectors convey the strength and direction of each trait's influence. DS, LWR, ind_rank, and CS are represented on the y-axis (PC2), while other variables are on the x-axis (PC1). Traits closer to the minimum angle indicate a positive correlation with that specific principal component (Fig. 5d), while those closer to the maximum angle suggest a negative correlation. This plot assists in identifying the associations between specific traits and the principal components, aiding in the interpretation of the reduced-dimensional space.

**Figure 6:**
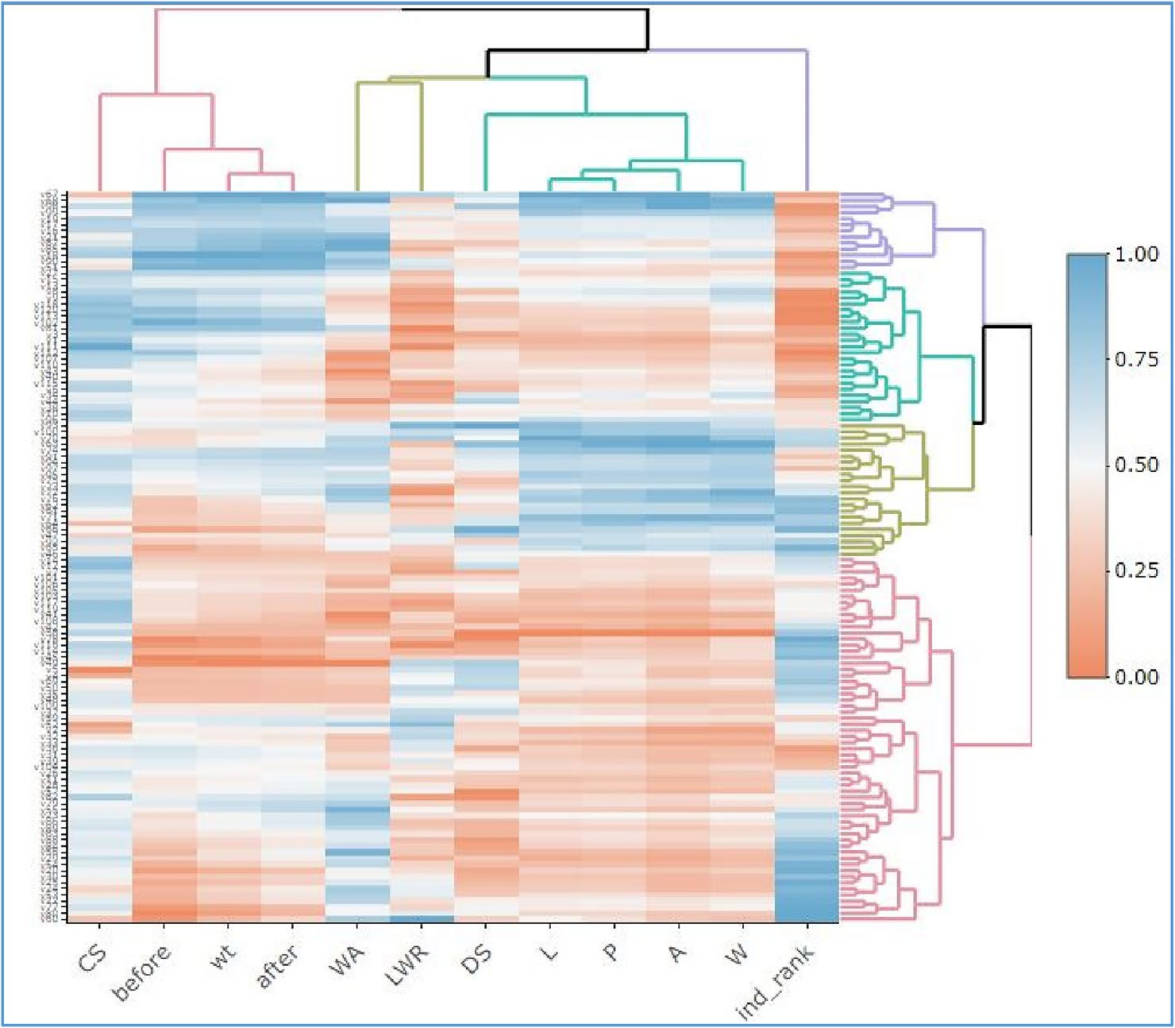
Heatmap of the whole standardized data unraveling the clustering of variables as well as genotypes based on similarity index.

The data visualization employs color-coded boxes representing z-scores (0 to 1) for each genotype based on various parameters (Fig. 6). The clustering of Group 1 genotypes is primarily attributed to the parameters "before," "wt" (weight), "after," "length" (L), "perimeter" (P), "area" (A), and "width" (W). These parameters exhibited a consistent range, from 0.75 to 1. The clustering of Group 1 genotypes also includes the "ind_rank" parameter, which falls within a range of 0.25 to 0. Group 2 genotypes exhibit clustering patterns associated with specific parameters. Within this group, parameters such as "CS", "before", "wt", and "after" consistently fall within the range of 0.75 to 1. In group 3 and 4 various other parameters also exhibited different ranges based on their clusters.

**Figure 7:**
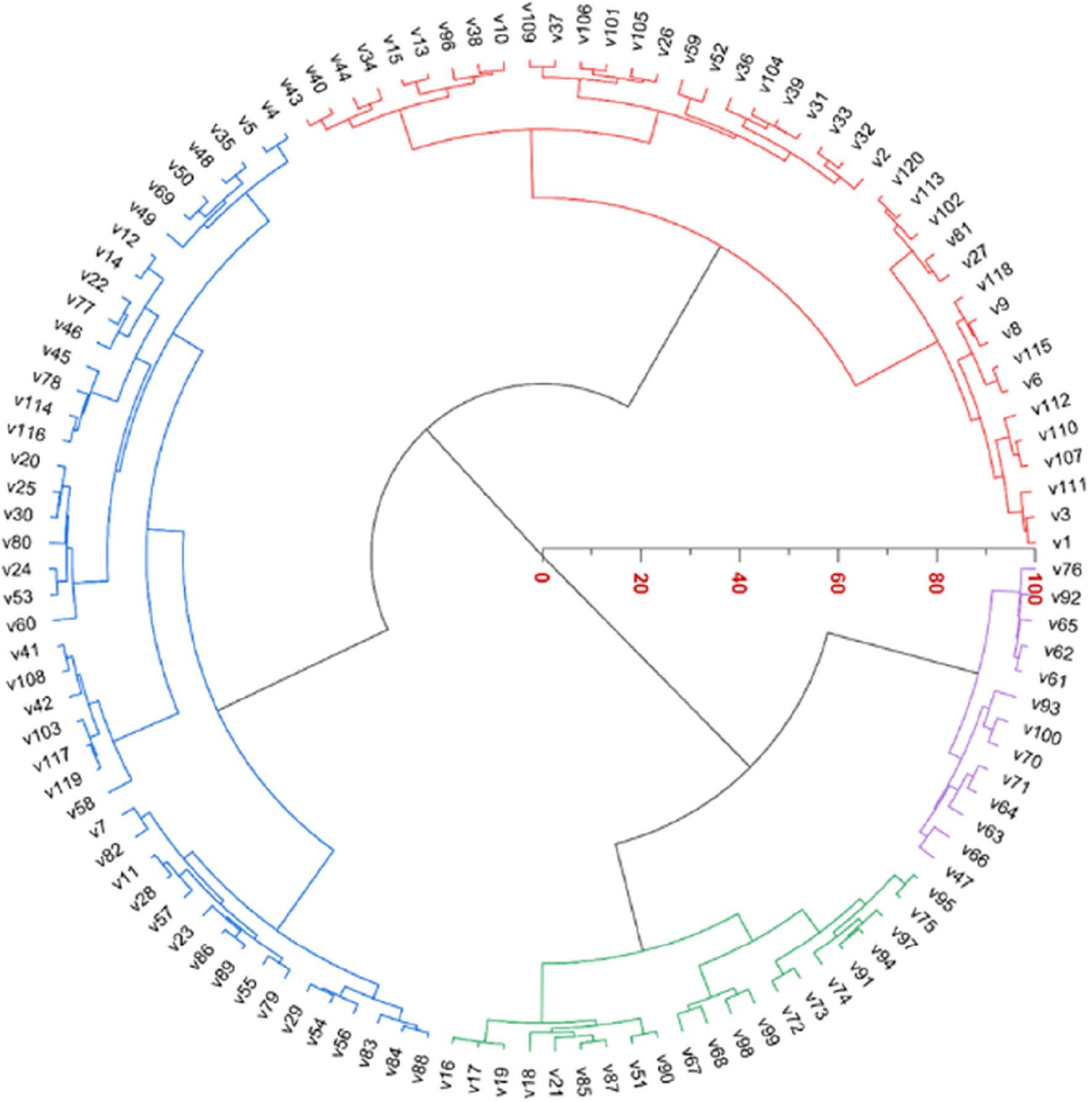
Cluster analysis.

Genotypes are grouped into clusters based on similarities (Fig. 7). Cluster 1, marked in red, comprises 40 genotypes (v1 to v43). In Cluster 2, represented in blue, 46 genotypes (v4 to v88) differ from those in Cluster 1. Cluster 3, depicted in green, includes 21 genotypes (v16 to v95) with dissimilarities to both Cluster 1 and Cluster 2. Cluster 4, in purple, consists of 13 genotypes distinctive from the other clusters. This clustering approach categorizes genotypes based on shared characteristics and dissimilarities, facilitating a clearer understanding of their relationships.

In the regression model, "wt," "W," "CS," and "DS" are independent variables. Coefficients (Coef) represent the change in the dependent variable for a one-unit change in the corresponding predictor variable. For example, in Table 4, a one-unit increase in "wt" is associated with a 0.2731 unit increase in the dependent variable, and this relationship is statistically significant (p-value < 0.001). Predictions about water absorption (WA) can be made based on these parameters, indicating that increasing "wt" leads to increased WA, while decreasing "CS" results in increased WA.Standard error (SE Coef) measures uncertainty in coefficient estimates. The t-value assesses whether a coefficient significantly differs from zero. P-values for all coefficients are below 0.001, signifying high statistical significance. Variation inflation factor (VIF) gauges multicollinearity, indicating how much the variance of coefficients is affected. Overall, the model suggests significant relationships between the independent variables and water absorption, providing insights into predictive factors and their statistical significance.

**Table 4:** A model summary with linear regression coefficients.

Coefficients						
Term	Coef	SE Coef	95% CI	T-value	P-value	VIF
Constant	0.4957	0.0895	(0.3185, 0.6730)	5.54	< 0.001	
Wt	0.2731	0.0444	(0.1851, 0.3610)	6.15	< 0.001	1.2
W	0.03662	0.0079	(0.02098, 0.05226)	4.64	< 0.001	1.52
CS	− 0.753	0.126	(− 1.003, − 0.504)	− 5.98	< 0.001	1.18
DS	− 0.1748	0.0387	(− 0.2514, − 0.0982)	− 4.52	< 0.001	1.48

Model summary						
S	R-sq	R-sq (adj)	PRESS	R-sq (pred)	AICc	BIC
0.03635	47.08%	45.24%	0.163327	43.12%	− 447.31	− 431.33

Analysis of variance						
Source	DF	Seq SS	Contribution (%)	Adj MS	F-value	P-value
Regression	4	0.13518	47.08	0.033794	25.58	< 0.001
wt	1	0.06756	23.53	0.049995	37.84	< 0.001
W	1	0.0111	3.87	0.028417	21.51	< 0.001
CS	1	0.0295	10.27	0.0472	35.72	< 0.001
DS	1	0.02702	9.41	0.027016	20.45	< 0.001
Error	115	0.15195	52.92	0.001321		
Total	119	0.28713	100.00			

Durbin-Watson Statistics = 1.002 | VIF: variation inflation factor; trait legends are same as in Table 1.

**Figure 8:**
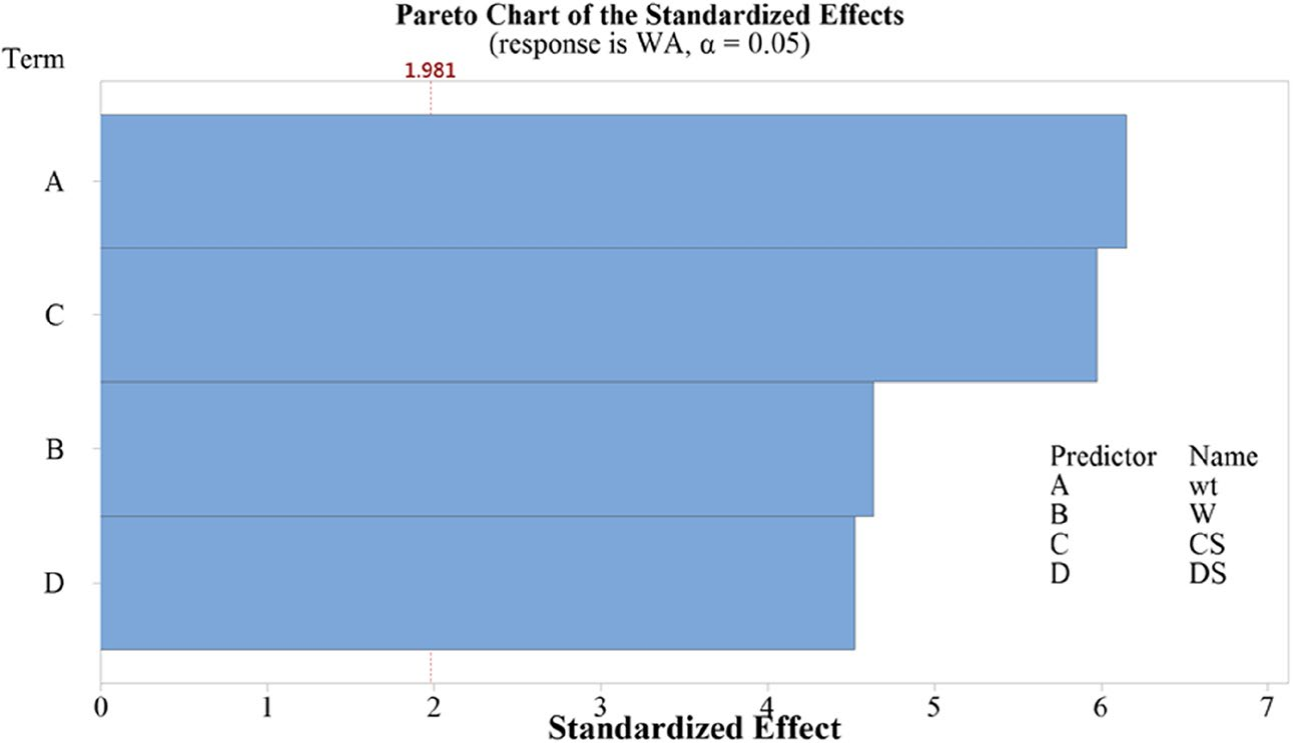
Effect of four traits on water absorption (WA) capability regressed on a panel of 120 genotypes. The value of 1.981 is the critical t-value above which the effect of predictor variable is significant (p-value < 0.001).

The Pareto chart (Fig. 8) analyzes the standardized effects of four traits on water absorption in 120 genotypes. The t-value, representing the ratio of the estimated effect to the standard error, was calculated for each trait. A critical t-value of 1.981 was used to assess statistical significance. In descending order on the Pareto chart, the standardized effects of predictors are as follows:

Predictor A (wt) has a significant standardized effect of approximately 6.2 on water absorption. Predictor C (CS) shows a substantial standardized effect of 6 on water absorption. Predictor B (W) demonstrates a notable standardized effect of 4.6 on water absorption. Predictor D (DS) displays a significant standardized effect of 4.5 on water absorption. All these effects are statistically significant at a very low p-value (less than 0.001), indicating their substantial impact on water absorption capability in the examined genotypes.

**Figure 9:**
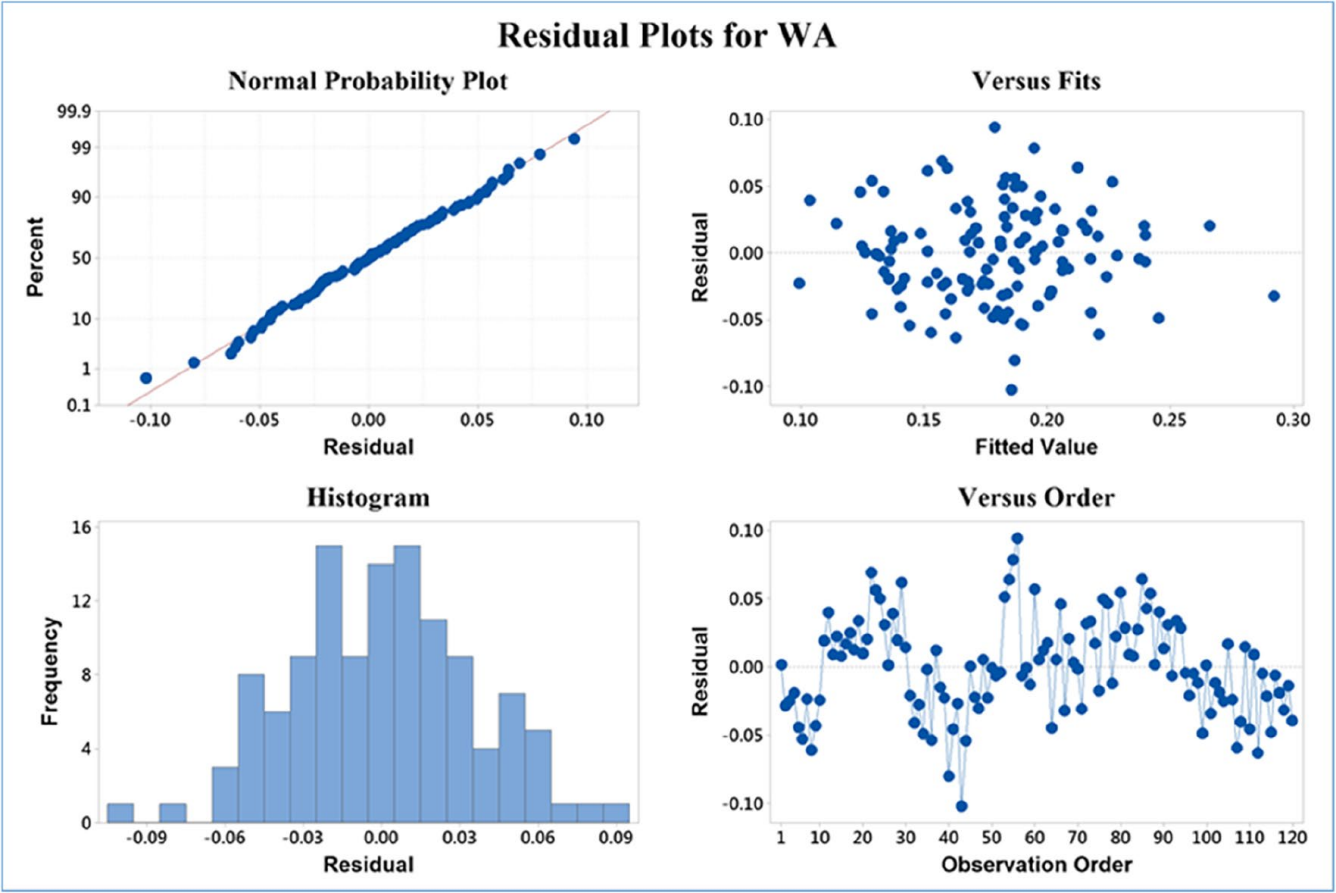
A summary of residual analysis as a result multiple linear regression.

A summary of residual in the ongoing regression model (Fig. 9) indicates that in a normal probability plot, residuals are plotted against percentiles, and a close alignment with the dotted line suggests a normal distribution of residuals. A residual and fitted value plot, assessing a regression model, depicts residuals on the y-axis and fitted values on the x-axis. Residuals are the differences between observed and predicted values. In a well-fitted model, residuals should scatter randomly around the zero line on the plot, indicating a good fit. In this case, the data points exhibit such random scattering, suggesting the regression model is well-fitted.

A histogram plot of residuals visually represents the distribution of differences between observed and predicted values in a regression model. The x-axis displays bins or intervals of residual values, while the y-axis shows the frequency count within each bin. A bell-shaped curve suggests a normal distribution, ideal for regression. Skewness indicates non-normal distribution. In the "versus order" plot, the x-axis represents the sequence of observations, and the y-axis shows residuals. Systematic patterns in this plot, such as trends, may suggest violations of model assumptions like non-linearity. In the provided data, the plot indicates systematic patterns, implying a potential violation of the assumption of linearity in the model.

## Discussion

Automated methods leveraging artificial vision and algorithms are commonly utilized for shape comparisons in various fields, including remote sensing and morphology. Within botany, these techniques find application in the analysis and classification of seeds across diverse taxonomic groups^26^. Seed shape analysis plays a crucial role in assessing wheat grain quality, as it can provide valuable information about seed size, symmetry, and uniformity. In recent years, advancements in technology have facilitated more accurate and efficient methods for seed shape analysis^2^. Enhancing grain shape and size has been a crucial focus in breeding efforts, driven by market and industry demands. The identification of genes that govern grain weight, size, and shape in wheat, along with the creation of functional markers, is essential for facilitating marker-assisted selection (MAS)^27^. Significant advancements in modern digital cameras and image processing algorithms have enabled accurate estimation of subtle differences in the color properties, shape, and size of seeds^28^. The application of digital image analysis to wheat seeds such as imbibition, environmental stress responses, predictive modeling etc. demonstrates its versatility in addressing various agricultural challenges, from breeding programs to quality control and environmental stress studies. In Descriptive Statistics, Various seed parameters before and after a treatment, measures such as means, standard errors (SE), minimums, maximums, quartile values (Q1 and Q3), percentage differences, t-values, and Cohen's d (effect size). The data indicate a significant increase in seed weight after treatment, with an impressive 35.47% difference. This substantial change, supported by a large Cohen's d of 3.46, underscores the treatment's effect on seed hydration. The seeds absorbed water during imbibition, leading to a significant increase in weight. Such hydration is vital for initiating germination, as it activates metabolic processes within the seed. In ANOVA analysis, factors such as Variety (V), Treatment (T), and their interaction (V x T) play a crucial role in understanding the variance in seed traits. Alongside these, the error component, grand mean (GM), and coefficient of variation percentage (CV%) are essential statistics. The analysis reveals that the majority of seed weight variation (41.80%) is attributed to the variety factor (V), with treatment (T) accounting for 48.76%. The interaction between variety and treatment (V x T) explains a smaller portion (4.38%), leaving a minimal residual error (5.06%). This indicates that both variety and treatment significantly contribute to the observed variability in seed weight. The iPASTIC indices provide a comprehensive evaluation of the genotypes, considering multiple seed traits. The use of Pearson's correlation coefficients is a valuable statistical tool for examining the relationships between different variables or traits. When combining the data from both before and after water treatment (n = 240), new correlations were calculated. However, this time, a more stringent significance level of p < 0.001 was used, and the degrees of freedom increased to 238. In the next analysis, a critical r-value of ± 0.297 was established, beyond which correlations were considered statistically significant at a high significance level of p < 0.001, with 118 degrees of freedom. Negative correlations below -0.297 that are also statistically significant at p < 0.001 suggest strong inverse relationships between the associated traits. When one trait increases, the other tends to decrease.

The PCA has yielded 12 Principal Components (PCs). Each Principal Component (PC) is associated with an eigenvalue. An eigenvalue represents the amount of variation in the original data that is explained by that particular PC. In this analysis, PC1 has an eigenvalue of 5.27, PC2 has an eigenvalue of 3.26, PC3 has an eigenvalue of 1.595, and PC4 has an eigenvalue of 1.2111. The heatmap appears to represent a comprehensive analysis of genotypes clustered into four distinct groups based on their z-scores, which are calculated from various parameters. Agglomerative Hierarchical Clustering is a bottom-up approach where smaller clusters are merged into larger ones based on their similarity. In this case, genotypes with similar trait patterns were successively merged into clusters until the four distinct clusters (C1, C2, C3, and C4) were formed.

The multiple linear regression analysis conducted here is valuable for understanding the relationships between specific traits (wt, W, CS, DS) and water absorption (WA). The Pareto chart helps researchers and analysts prioritize their efforts by identifying the most influential traits affecting water absorption capability among the genotypes.

A wheat seed's ability to absorb water does not primarily determine its drought tolerance. However, it is a factor. Drought resistance in wheat is comprised of a variety of genetic and physiological features that allow the plant to withstand or tolerate periods of inadequate water supply. A seed's ability to absorb water is connected to its vigor and ability to germinate under favorable conditions. A seed that can efficiently absorb water may have an advantage during germination, but this does not guarantee drought tolerance.

Wheat seed morphology may not be a direct indicator of drought tolerance. However, certain seed morphometric traits may be related to drought tolerance to some extent. Seeds with larger sizes or plumper forms, for example, may store more reserve nutrients, perhaps giving seedlings an early edge during germination and early growth under water limited conditions. This could be beneficial in drought-prone places since it gives the seedling a better opportunity of establishing itself. The future value of wheat seed shape analysis lies in its potential to drive advancements in agriculture, genetics, and technology. As researchers continue to explore and apply novel techniques, the insights gained from seed shape analysis will contribute to more sustainable, resilient, and productive wheat farming practices.

Furthermore, some research suggests that certain seed morphologies may be linked to distinct genetic features related to drought resistance. However, the association between seed shape and drought resistance is complex and varies based on a range of factors such as wheat type, environmental conditions, and other genetic features implicated in drought adaptation. In short, while seed shape may have some indirect connections with drought resistant features, it is not a definitive or exclusive indication. Drought tolerance in wheat is a multidimensional feature influenced by genetic and physiological factors other than seed shape.

## Conclusion

In conclusion, the digital image analysis of seeds from 120 wheat lines in relation to water-absorbing capacity has provided valuable insights into the complex dynamics of seed imbibition. Through advanced image processing techniques and comprehensive statistical analysis, we have gained a deeper understanding of how wheat seeds transform during the early stages of germination. Our study highlighted the significant role of water absorption in seed development and germination. We observed substantial changes in seed size, weight, shape, and other characteristics as seeds absorbed water. These transformations are fundamental to the initiation of metabolic processes required for germination. The connection between research findings in wheat seed shape analysis and their implications for crop improvement is a dynamic and multifaceted relationship. Bridging the gap between scientific insights and on-the-ground applications is essential for realizing the full potential of seed shape analysis in driving sustainable and resilient wheat cultivation.

In summary, our research underscores the importance of digital image analysis in unraveling the dynamics of seed imbibition in wheat. These findings contribute to our knowledge of seed development, germination, and the critical role of water absorption. The insights gained from this study have practical applications in agriculture, breeding, and crop improvement, ultimately contributing to more resilient and productive wheat crops. The integration of advanced analytical techniques with rigorous statistical methods has strengthened the validity and significance of our findings in this critical area of agricultural research.

## Data Availability

All data generated or analyzed during this study are included in this published article.
